# Investigating the prognostic value of mTORC1 signaling in bladder cancer via bioinformatics evaluation

**DOI:** 10.1038/s41598-023-49366-w

**Published:** 2023-12-12

**Authors:** Xin Yu, Wenge Li, Shengrong Sun, Juanjuan Li

**Affiliations:** 1 Department of Breast and Thyroid Surgery, Renmin Hospital of Wuhan University, 238 Ziyang Road, Wuhan, 430060 Hubei Province People’s Republic of China; 2 Department of Oncology, Shanghai Artemed Hospital, Shanghai, People’s Republic of China; 3 Department of General Surgery, Taikang Tongji (Wuhan) Hospital, 322 Sixin North Road, Wuhan, 430050 Hubei Province People’s Republic of China

**Keywords:** Bladder cancer, Cancer metabolism, Tumour biomarkers, Tumour immunology

## Abstract

Bladder cancer, a prevalent and heterogeneous malignancy, necessitates the discovery of pertinent biomarkers to enable personalized treatment. The mammalian target of rapamycin complex 1 (mTORC1), a pivotal regulator of cellular growth, metabolism, and immune response, exhibits activation in a subset of bladder cancer tumors. In this study, we explore the prognostic significance of mTORC1 signaling in bladder cancer through the utilization of bioinformatics analysis. Our investigation incorporates transcriptomic, somatic mutation, and clinical data, examining the mTORC1 score of each sample, as well as the enrichment of differentially expressed genes (DEGs), differentiation characteristics, immunological infiltration, and metabolic activity. Our findings reveal that elevated mTORC1 levels serve as an adverse prognostic indicator for bladder cancer patients, exhibiting a significant association with Basal-type bladder cancer. Patients with heightened mTORC1 activation display heightened levels of pro-carcinogenic metabolism. Additionally, these individuals demonstrate enhanced response to immunotherapy. Finally, we develop an mTORC1-related signature capable of predicting the prognosis of bladder cancer patients.The signature offers novel mTORC1-related biomarkers and provides fresh insights into the involvement of mTORC1 in the pathogenesis of bladder cancer.

## Introduction

Bladder cancer is a prevalent malignancy that poses a significant challenge regarding cancer-related morbidity and mortality worldwide^1^. Despite improvements in treatment options, the disease remains difficult to manage due to its high recurrence and metastasis rates and heterogeneity. Hence, it is essential to identify pertinent biomarkers that have a predictive significance for treatment response and clinical consequences in an attempt to develop personalized therapeutic approaches^2^.

The mammalian target of rapamycin complex 1 (mTORC1) is a highly conserved serine/threonine kinase, which exerts crucial effects on regulating multiple fundamental cellular processes. These cellular processes include but are not limited to cell growth, proliferation, metabolism, and autophagy, which are critical for the proper functioning of cells^3,4^. mTORC1 acts as an essential regulator of cellular energy and nutrient balance, serving as a vital sensor of intracellular nutrient and energy status. By integrating and interpreting signals from various sources, including growth factors, amino acids, glucose, and oxygen, mTORC1 orchestrates critical cellular processes such as cell growth, proliferation, maintenance of homeostasis, and cell death^5–8^. It is composed of several essential components that work in coordination to regulate the activity of the complex. The core component of the complex is the mTOR kinase, which is responsible for the phosphorylation of downstream targets. Other critical components include the regulatory-associated protein of mTOR (Raptor), which contributes to recruiting substrates to mTORC1, and mammalian lethal with Sec13 protein 8 (mLST8), which is involved in stabilizing the complex structure. Additionally, the complex contains a proline-rich Akt substrate of 40 kDa (PRAS40), a negative regulator that inhibits mTORC1 activity until it is phosphorylated by Akt, and DEP-domain-containing mTOR-interacting protein (DEPTOR), another negative regulator that binds to mTORC1 to inhibit its activity^6^.

When activated by signals from growth factors or nutrients, mTORC1 triggers the synthesis of proteins in cells by activating a few downstream molecules like S6 kinase 1 (S6K1) and eukaryotic translation initiation factor 4E-binding protein 1 (4EBP1) through phosphorylation. This process contributes to regulating cell growth and metabolism^9^. mTORC1 also modulates the tumor microenvironment by regulating angiogenesis, inflammation, and immune response, which in turn promotes tumor progression and metastasis^3,4,9^. In bladder cancer, mTORC1 signaling is activated in a subset of tumors and is associated with poor prognosis^10^. Despite extensive research on the role of mTORC1 in cancer, its precise functional contribution to bladder cancer remains unclear.

This research aims to examine the physical and genetic attributes of bladder cancer with mTORC1 signaling and assess how this may affect the patient's overall health and the surrounding immune system. Additionally, we aim to develop and validate a prognostic signature based on mTORC1 signaling pathway activity, which could serve as a valuable tool for individualized bladder cancer management.

## Materials and methods

### Data processing

**Figure 1:**
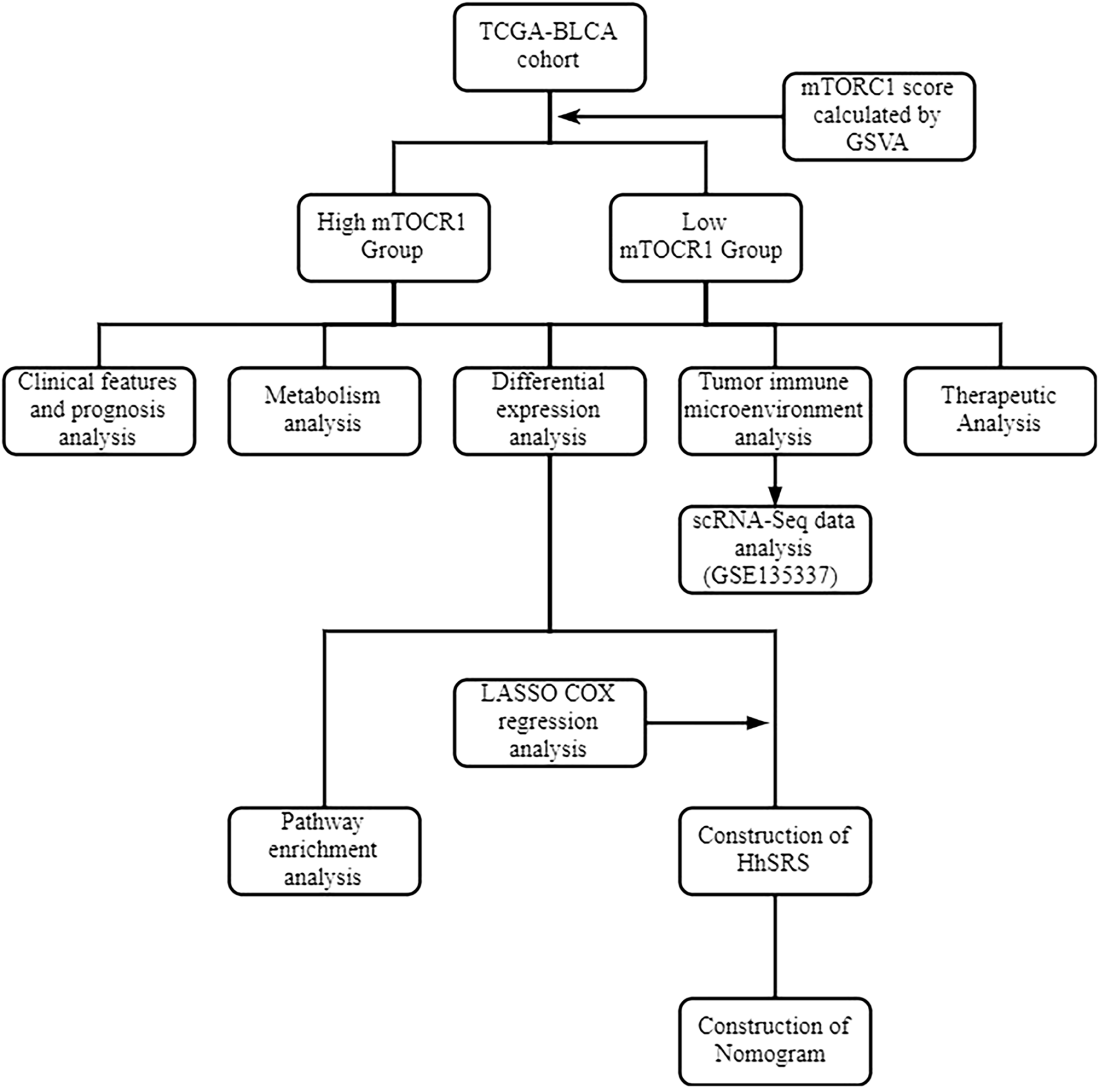
The framework design of the study.

The study's framework design is illustrated in Fig. 1. We acquired RNA sequencing (RNA-seq) information from 408 patients with bladder cancer by obtaining fragments per kilobase of exon per million mapped fragments (FPKM) values and matching them with clinical data from The Cancer Genome Atlas (TCGA) through the Xena data portal of UCSC. After retrieving the RNA-seq data, the FPKM values were depicted as the transcripts per million (TPM). To analyze somatic mutation data, we utilized the R package TCGAbiolinks^11^ to obtain the data in the Mutation Annotation Format (MAF), which was further analyzed by using the R package maftools. For the single-cell RNA-seq (scRNA-seq) analysis, a dataset of bladder cancer (GSE1353379^12^) was utilized, which contained scRNA-seq data. Furthermore, the R package IMvigor210CoreBiologies^13^ was used to obtain comprehensive expression data and clinical information for 348 bladder cancer patients who underwent atezolizumab treatment.

### Computation of enrichment scores of gene signatures

Utilizing transcriptomic data, we employed a nonparametric and unsupervised method called Gene Set Variation Analysis (GSVA) to predict the activity of specific pathways^14^. We obtained the gene signature for mTORC1 signaling from the essential gene set collection in the Molecular Signatures Database (MsigDB) through their online platform (https://www.gsea-msigdb.org/gsea/downloads.jsp). We collected various gene signatures for bladder cancer research, including molecular subtype-specific signatures^15^, metabolism gene sets from KEGG database collections^16^ in MsigDB, and drug-specific gene signatures from the study of Hu et al*.*^17^. Some of these signatures are associated with pathways that are inhibited by the immune system, while others are linked to targeted therapies that have been developed for specific genetic mutations. Additionally, there are gene signatures that predict how well a patient may respond to radiotherapy. All gene sets can be found in Supplementary Table S1.

### Screening and functional annotation of differentially expressed genes (DEGs)

Limma R package was applied to identify DEGs that were either upregulated or downregulated with *p* < 0.05 and a fold change (FC) > 3/2 as screening criteria. We then utilized the R package ClusterProfiler to perform Gene Ontology (GO), Kyoto Encyclopedia of Genes and Genomes (KEGG), and hallmark gene set enrichment analysis. Additionally, we performed gene set enrichment analysis (GSEA) using the same R package to examine gene sets that were significantly enriched.

### Identification of bladder cancer subtypes based on molecular characteristics

We utilized the ConsensusMIBC R package to classify bladder cancer into different molecular subtypes based on various molecular subtype systems such as TCGA, MDA, Baylor, CIT, Lund, and UNC subtypes.

### Evaluation of immune cell infiltration

To analyze the immune microenvironment of bladder cancer, we utilized CIBERSORT, a tool that evaluates the proportions of 22 distinct immune cell types that infiltrate into the tumors in each sample. We also obtained cancer immunity cycle gene sets from a previous study conducted by Xu et al.^18^ and calculated the gene set enrichment score using GSVA. This allowed us to measure the activity of genes involved in the different stages of the cancer immunity cycle in each sample.

### Chemotherapy response prediction

We estimated the half-maximal inhibitory concentration (IC50) of common chemotherapeutic agents using the "pRRophetic" R package. Additionally, we screened the drug-target genes using the Drugbank database^19^.

### Construction of the mTORC1-related signature (mTORC1RS) and the mTORC1RS-related nomogram

The mTORC1RS was developed using data from the TCGA-BLCA study. Genes with differential expression levels in low and high mTORC1 signaling groups were identified using univariate Cox regression analysis to determine genes associated with overall survival (OS). The candidate genes were further analyzed using LASSO regression to select the most relevant ones for building the mTORC1RS model. LASSO regression was then employed to select the most important genes. Thereafter, the multiple regression model and the regression coefficients of the survival-associated genes were determined by multivariate Cox regression analysis. The mTORC1RS was developed using a mathematical formula, which is as follows: Risk score = Σn1 coefi*xi. Using the expression levels of the identified genes, we computed the individual patient score for risk based on a specific formula. The risk score was utilized to classify patients into high-risk or low-risk groups using the mTORC1RS. The median risk score was used as a threshold to divide patients into two groups, with those above the threshold classified as high-risk and those below classified as low-risk.

To find possible independent indicators of prognosis, multivariate Cox analysis was performed on both the mTORC1RS grouping and clinical variables. After that, a nomogram related to mTORC1RS was constructed utilizing the regplot software, with age, stage, and mTORC1RS group as parameters. This nomogram can provide an estimation of survival probability for bladder cancer patients based on their age, cancer stage, and mTORC1RS group.

### scRNA-seq data analysis

We used the Seurat R package to perform unsupervised clustering of individual cells based on the read count matrix as input. Data quality control, cell clustering, and annotation were executed based on the scRNA-seq data as previously described^20^. For intercellular communication network-related analysis, the iTalk R package was employed.

### Statistical analysis

To investigate the relationships between different variables, Pearson correlation analysis was employed. For continuous variables that conform to a normal distribution, a *t*-test was conducted to compare them among binary groups. The Kruskal–Wallis test was conducted to evaluate differences among more than two groups. To determine whether there were statistically significant differences between the subgroups in each dataset, the log-rank test was utilized, and Kaplan–Meier (KM) method was used to generate survival curves. All statistical analyses were conducted using SPSS 22.0, SangerBox^21^, and R 4.0.0. The *p* values obtained were two-tailed, and values less than 0.05 were taken as evidence of statistical significance.

## Results

### mTORC1 signaling in bladder cancer

**Figure 2:**
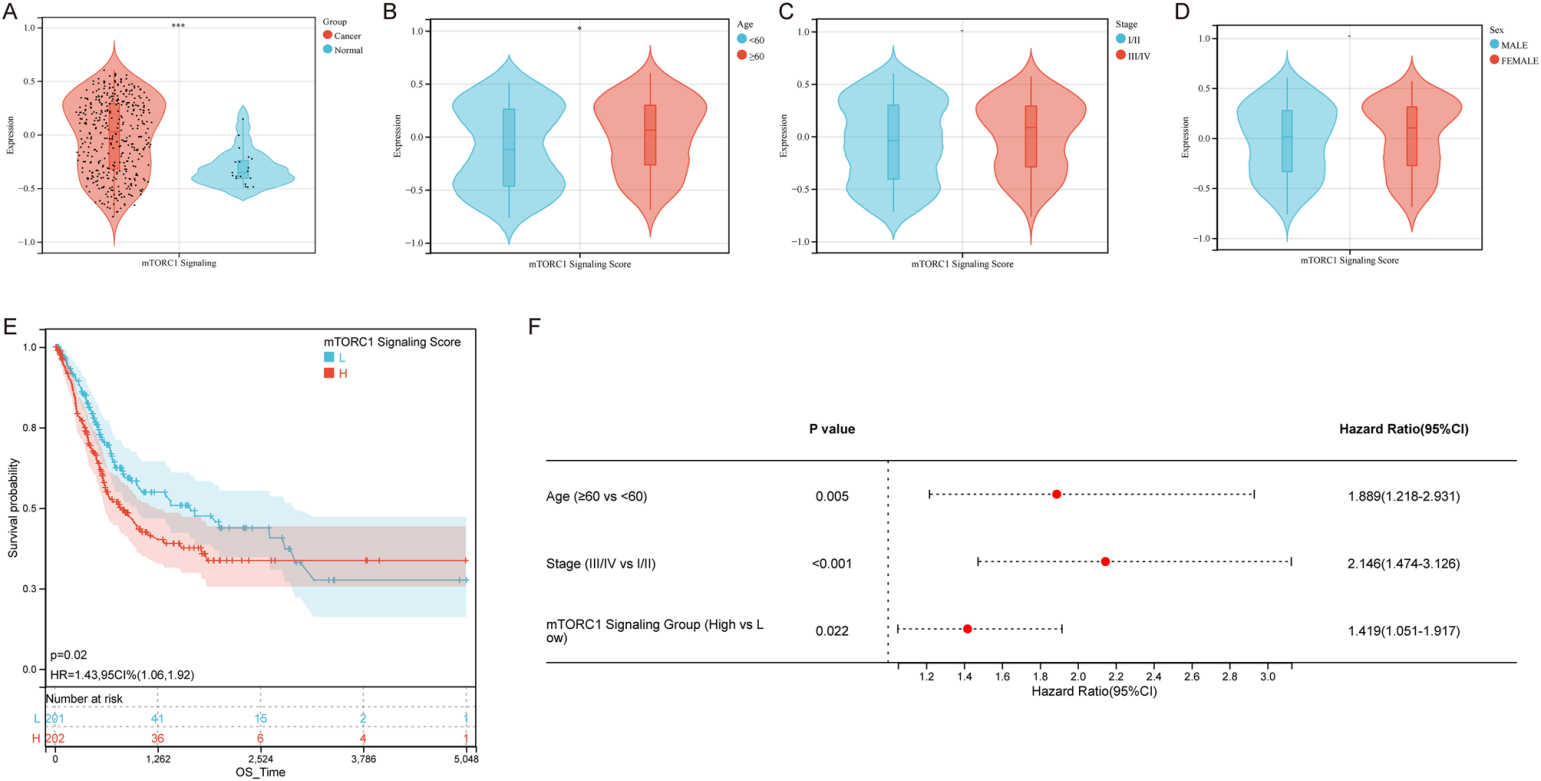
High mTORC1 signal in bladder cancer suggests poor prognosis. (**A**) Comparison of mTORC1 scores between bladder cancer tissues and adjacent tissues. (**B**–**D**) Analysis of mTORC1 scores in different subgroups based on (**B**) age, (**C**) stage, and (**D**) sex. (**E**) Kaplan–Meier analysis and (**F**) Cox regression analysis of mTORC1 scores in overall survival (OS) of bladder cancer patients. − *P *> 0.05, *P < 0.05, **P < 0.01, ***P < 0.001, and ****P < 0.0001 indicate statistical significance.

Initially, we assessed the mTORC1 signaling scores of both normal and bladder cancer samples. We analyzed the TCGA dataset and observed that the mTORC1 signaling score in normal tissues was considerably lower than that in breast cancer tissue samples (Fig. 2A). We analyzed the expression of 200 mTORC1 signaling genes and demonstrated that 54% (108/200) of genes displayed high expression level in bladder cancer samples, while 17.5% (35/200) showed low expression levels (Supplementary Fig. S1). In addition, we investigated the association between clinical factors and mTORC1 signaling in bladder cancer patients. Our results indicated that mTORC1 signaling was significantly overactivated in older patients (Fig. 2B). However, we did not observe any significant difference in mTORC1 signaling scores between patients of different stages and genders (Fig. 2C,D).

After calculating the mTORC1 signaling scores for all patients, they were divided into two groups, one with high scores and the other with low scores, using the median score (Supplementary Fig. S2). The high mTORC1 group showed less favorable clinical outcomes compared to the low mTORC1 group, as revealed by the KM analysis (Fig. 2E). Additionally, the multivariate Cox regression analysis revealed that mTORC1 signaling was a significant independent predictor of prognosis in patients with bladder cancer (Fig. 2F).

### Identification of DEGs and their functional annotations

**Figure 3:**
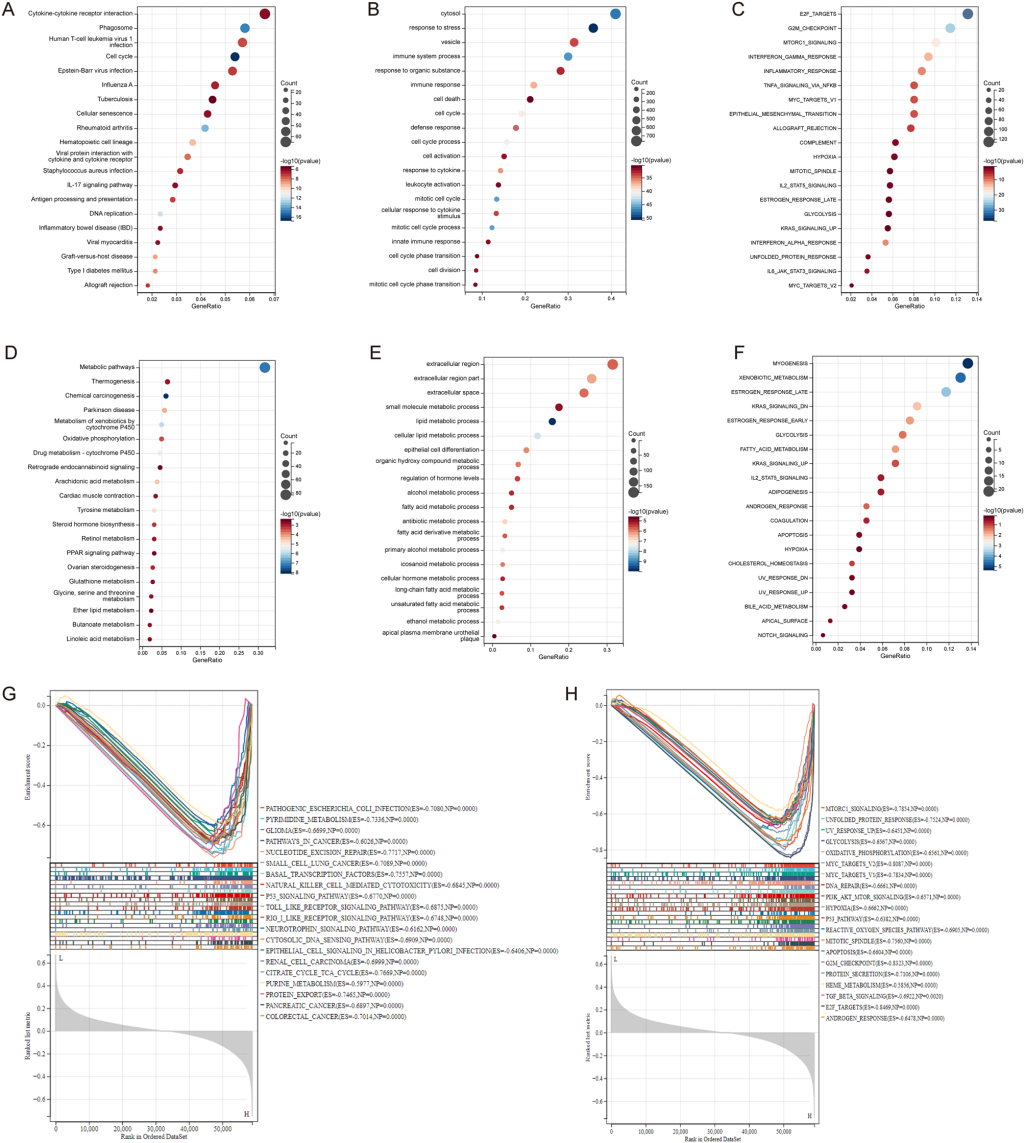
Functional annotation of mTORC1 signaling. (**A**–**C**) Enrichment analysis of upregulated differentially expressed genes (DEGs) in (**A**) KEGG, (**B**) GO, and (**C**) Hallmark pathways. (**D**–**F**) Enrichment analysis of downregulated DEGs in (**D**) KEGG, (**E**) GO, and (**F**) Hallmark pathways. (**G**, **H**) Functional analysis of mTORC1 using gene set enrichment analysis (GSEA) based on (**G**) KEGG and (**H**) Hallmark gene sets.

After identifying the DEGs, we found that 2044 genes were upregulated and 957 genes were downregulated in the high mTORC1 signaling group (Supplementary Fig. S3A, B). To further understand the biological functions of these DEGs, we conducted functional annotations using the KEGG analysis. Our results indicated that the upregulated DEGs were primarily associated with "Cytokine-cytokine receptor interaction," "Phagosome," and "Human T-cell leukemia virus 1 infection" pathways (Fig. 3A). After identifying the DEGs, we performed a GO analysis to explore the biological activities linked to the upregulated DEGs. The results revealed that these genes were significantly enriched in "Cytosol," "Response to stress," and "Vesicle" (Fig. 3B). Furthermore, a hallmark gene sets analysis was performed, which indicated that the "E2F Targets," "G2M Checkpoint," and "mTORC1 signaling" pathways were enriched among the DEGs (Fig. 3C). In terms of the downregulated DEGs, the analysis of KEGG pathway indicated that these genes were mostly involved in "Metabolic pathways," "Thermogenesis," and "Chemical carcinogenesis" (Fig. 3D). The GO analysis revealed that the highly enriched pathways were related to the "Extracellular region," "Extracellular region part," and "Extracellular space" (Fig. 3E). Additionally, the hallmark gene sets analysis indicated that "Myogenesis," "Xenobiotic metabolism," and "Estrogen response late" were enriched in these downregulated DEGs (Fig. 3F).

To further investigate the association between mTORC1 signaling and bladder cancer, we conducted a GSEA and found that patients with elevated mTORC1 signaling had a higher enrichment of the KEGG pathways "Pathogenic Escherichia coli infection," "Pyrimidine metabolism," and "Glioma," as well as the hallmark gene sets "mTORC1 signaling," "Unfolded protein response," and "UV response" (Fig. 3G,H). These findings suggest that mTORC1 signaling plays a significant role in bladder carcinogenesis by regulating metabolism and immunity.

### Association between mTORC1 signaling and molecular characteristics

**Figure 4:**
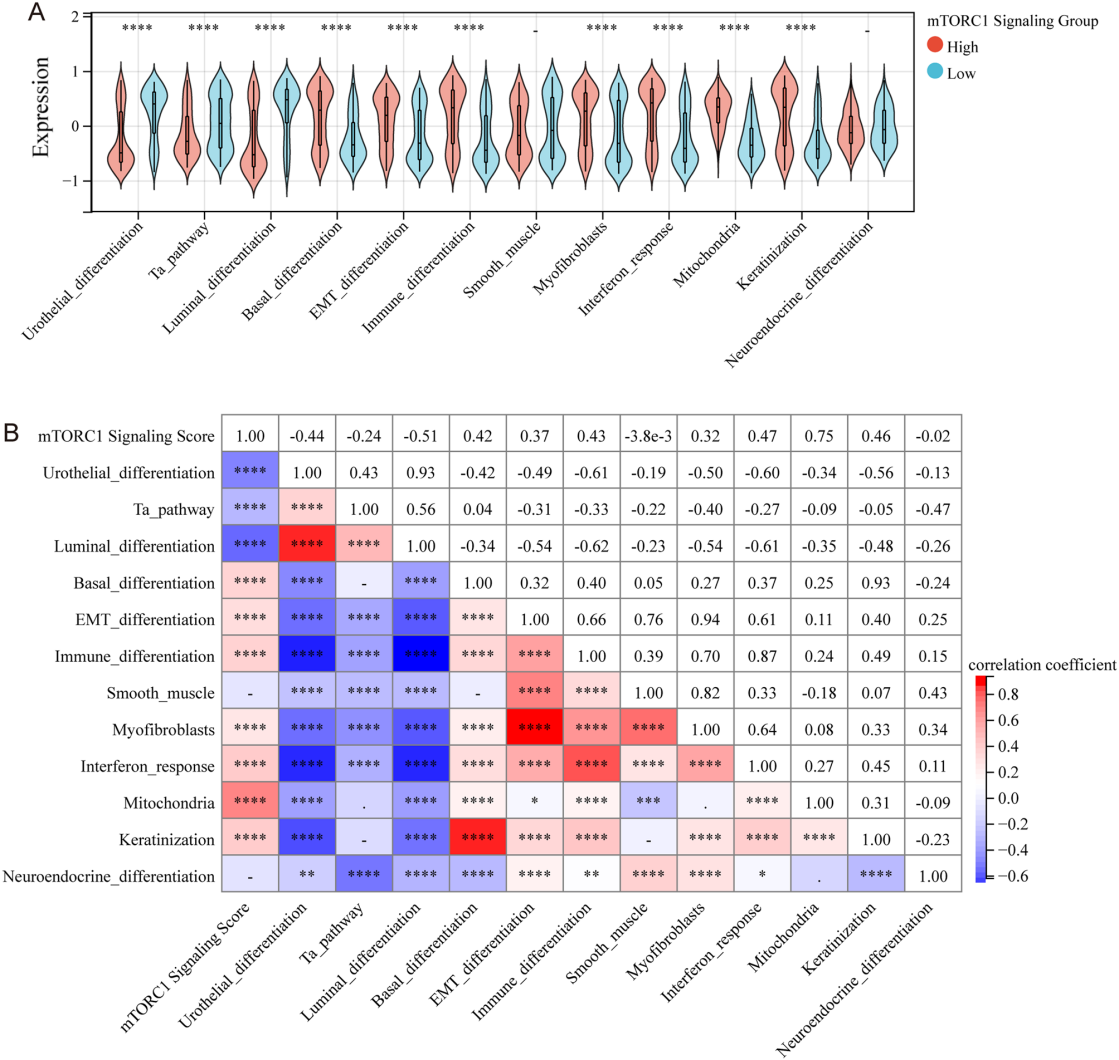
Correlation analysis between mTORC1 and molecular subtype in bladder cancer. (**A**) Differential expression of specific bladder cancer-related signatures in patients with high and low mTORC1 scores. (**B**) Correlation heatmap of specific bladder cancer-related signatures in patients with mTORC1 scores. − *P *> 0.05, *P < 0.05, **P < 0.01, ***P < 0.001, and ****P < 0.0001 indicate statistical significance.

Bladder cancer is known to have distinct molecular subtypes that have different prognoses and responses to different treatments, including immunotherapy and chemotherapy^15,22^. The study investigated the correlation between mTORC1 signaling and molecular subtypes of bladder cancer (Table 1). The analysis demonstrated that mTORC1 signaling was highly expressed in the basal-type bladder cancer subtype, as identified by six distinct computational models for predicting molecular subtypes. These findings suggest that targeting mTORC1 signaling may be an encouraging therapeutic option for patients with basal-type bladder cancer. In addition, a detailed molecular analysis of patients with high mTORC1 signaling showed higher basal differentiation, epithelial-mesenchymal transition (EMT), immune differentiation, myofibroblasts, interaction response, mitochondria, and keratinization scores compared to patients with low mTORC1 signaling. On the other hand, urinary differentiation, Ta pathway, and luminal differentiation scores were lowered in patients with high mTORC1 signaling (Fig. 4A,B). These results suggest that mTORC1 signaling is associated with specific molecular characteristics in bladder cancer.

**Table 1:** Correlations between mTORC1 and molecular subtypes using six different algorithms and bladder cancer signatures.

Characteristics	Low (N = 204)	High (N = 204)	Total (N = 408)	*p* value
Baylor.subtype				1.3e−21
Basal	25 (6.13%)	118 (28.92%)	143 (35.05%)	
Differentiated	179 (43.87%)	86 (21.08%)	265 (64.95%)	
UNC.subtype				1.2e−19
Basal	44 (10.78%)	136 (33.33%)	180 (44.12%)	
Luminal	160 (39.22%)	68 (16.67%)	228 (55.88%)	
CIT.subtype				3.2e−28
MC1	116 (28.43%)	46 (11.27%)	162 (39.71%)	
MC2	20 (4.90%)	5 (1.23%)	25 (6.13%)	
MC3	8 (1.96%)	10 (2.45%)	18 (4.41%)	
MC4	34 (8.33%)	5 (1.23%)	39 (9.56%)	
MC5	1 (0.25%)	0 (0.0e + 0%)	1 (0.25%)	
MC6	2 (0.49%)	2 (0.49%)	4 (0.98%)	
MC7	23 (5.64%)	136 (33.33%)	159 (38.97%)	
Lund.subtype				5.0e−19
Ba/Sq	4 (0.98%)	49 (12.01%)	53 (12.99%)	
Ba/Sq-Inf	9 (2.21%)	35 (8.58%)	44 (10.78%)	
GU	20 (4.90%)	12 (2.94%)	32 (7.84%)	
GU-Inf	15 (3.68%)	14 (3.43%)	29 (7.11%)	
Mes-like	16 (3.92%)	18 (4.41%)	34 (8.33%)	
Sc/NE-like	4 (0.98%)	13 (3.19%)	17 (4.17%)	
Uro-Inf	21 (5.15%)	2 (0.49%)	23 (5.64%)	
UroA-Prog	66 (16.18%)	28 (6.86%)	94 (23.04%)	
UroB	7 (1.72%)	17 (4.17%)	24 (5.88%)	
UroC	42 (10.29%)	16 (3.92%)	58 (14.22%)	
MDA.subtype				2.8e−23
Basal	24 (5.88%)	119 (29.17%)	143 (35.05%)	
Luminal	88 (21.57%)	57 (13.97%)	145 (35.54%)	
p53-like	92 (22.55%)	28 (6.86%)	120 (29.41%)	
TCGA.subtype				4.2e−23
Basal_squamous	20 (4.90%)	113 (27.70%)	133 (32.60%)	
Luminal	34 (8.33%)	12 (2.94%)	46 (11.27%)	
Luminal_infiltrated	51 (12.50%)	16 (3.92%)	67 (16.42%)	
Luminal_papillary	95 (23.28%)	51 (12.50%)	146 (35.78%)	
Neuronal	4 (0.98%)	12 (2.94%)	16 (3.92%)

### Correlations between mTORC1 signaling and metabolism pathways

**Figure 5:**
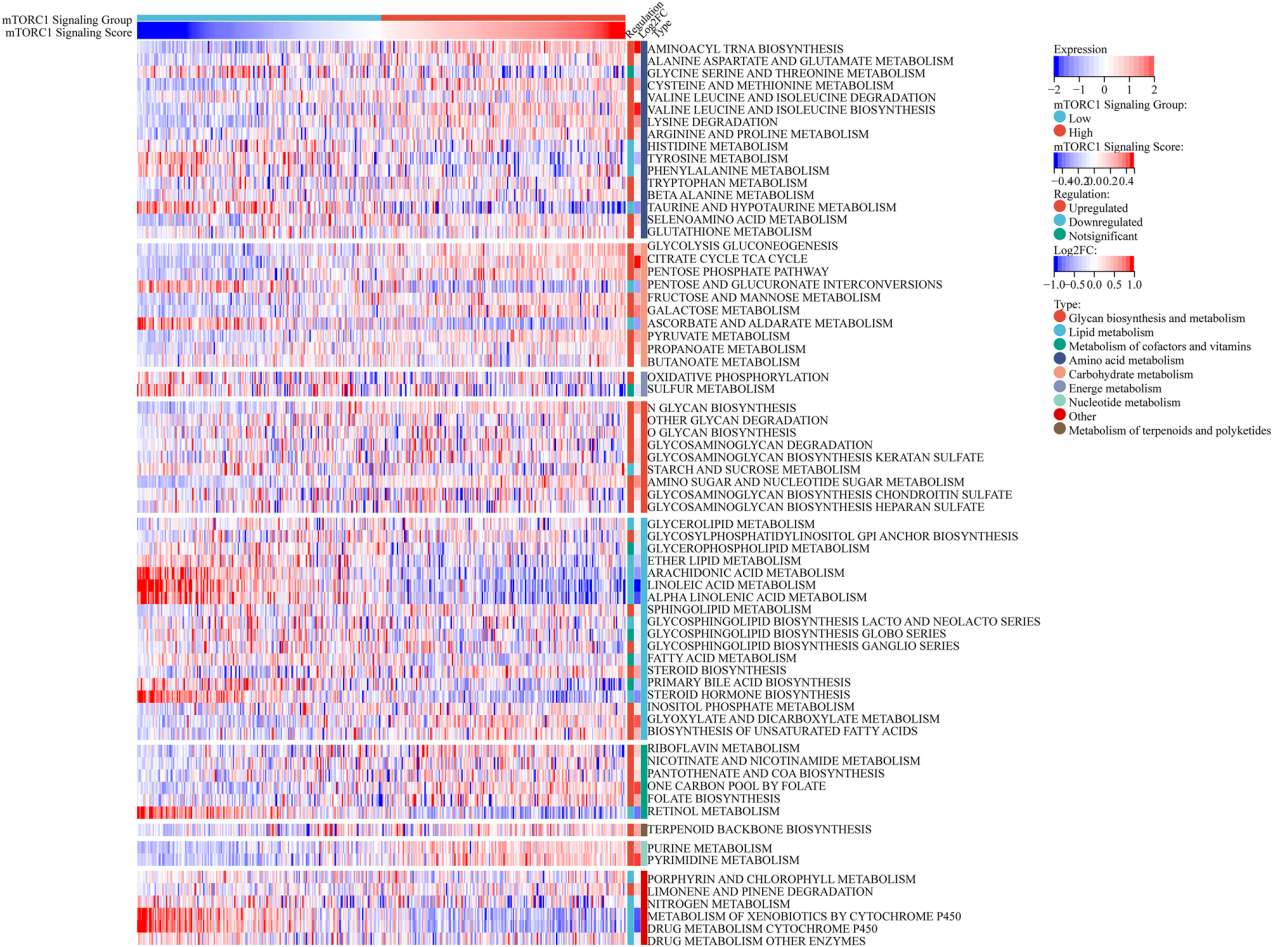
Correlation analysis between mTORC1 and bladder cancer metabolism. Heatmap showing the expression patterns of metabolism signatures in patients with high and low mTORC1 scores.

The study also investigated the association between mTORC1 signaling and metabolic pathways. The analysis revealed that patients with high mTORC1 signaling had significantly elevated metabolic activity (Fig. 5). Among the 70 KEGG metabolic pathways examined, 44 were upregulated, while 20 were downregulated in patients with high mTORC1 signaling. Moreover, patients with high mTORC1 signaling displayed increased activation of glycolysis, the pentose phosphate pathway, and nucleotide metabolic processes. These metabolic pathways have been known to promote malignant tumor progression, suggesting that the activation of mTORC1 signaling may be involved in the metabolic reprogramming of bladder cancer cells^23^. It was observed that patients with high mTORC1 signaling exhibited downregulation of linoleic acid metabolism and arachidonic acid metabolism, which have been previously reported to have anti-cancer effects^24,25^. Moreover, pathways associated with drug metabolism, such as "Cytochrome P450-mediated metabolism of xenobiotics" and "Drug metabolism through cytochrome P450", were also inhibited in this group. These findings suggest that drugs targeting these pathways may have superior efficacy in patients with high mTORC1 signaling.

### mTORC1 signaling association with tumor immune microenvironment (TIME)

**Figure 6:**
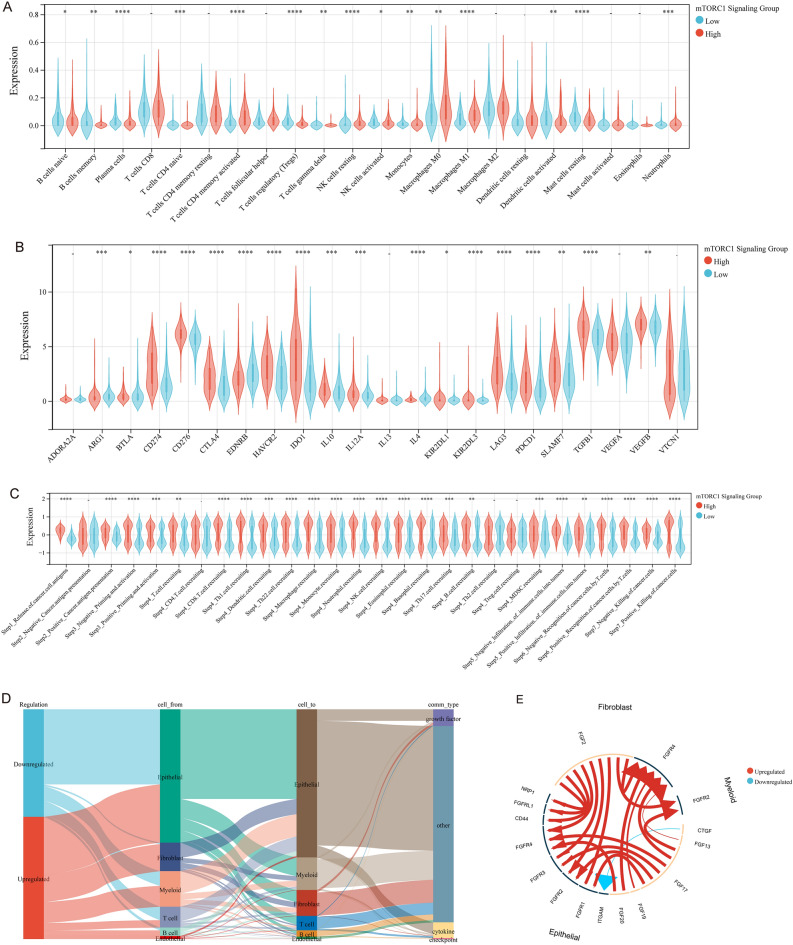
Correlation analysis between mTORC1 and tumor immune microenvironment (TIME). (**A**–**C**) Differential expression of (**A**) immune cells, (**B**) immune checkpoints, and (**C**) immune cycle score in patients with high and low mTORC1 scores. (**D**) Sankey diagram and (**E**) circos plots depicting ligand-receptor interactions with significant expression differences in the TIME of bladder cancer. − *P *> 0.05, *P < 0.05, **P < 0.01, ***P < 0.001, and ****P < 0.0001 indicate statistical significance.

To examine the impact of mTORC1 signaling on immunological features of the TIME, we assessed the presence of immune cells, evaluated the effectiveness of the cancer immunity cycle, and examined the expression of immune checkpoint genes (Fig. 6). We used the CIBERSORT algorithm to estimate the infiltration of immune cells in the TIME, and found that patients exhibiting high mTORC1 signaling had less Treg infiltration but higher infiltration of T cells CD4 memory activated, resting NK cells, and M1-like macrophages (Fig. 6A, Supplementary Fig. S4A). Although the proportion of infiltrated CD8^+^ T cells was slightly higher in the high mTORC1 signaling group, this was not statistically significant. Interestingly, the high mTORC1 signaling group had higher expression of immune checkpoint-related genes (Fig. 6B, Supplementary Fig. S4B). Concurring with these observations, the analysis of the immunity cycle revealed that mTORC1 signaling was positively correlated with scores of positive and negative regulation of most immune cycle steps (Fig. 6C, Supplementary Fig. S4C). To investigate further the impact of mTORC1 signaling in the TIME, we analyzed scRNA-seq data from seven bladder cancer patients (GSE135337) (Fig. Supplementary S4D). These samples were then partitioned into high and low mTORC1 signaling groups based on the average mTORC1 signaling score expression of the samples (Fig. Supplementary S4E). Using iTalk to deduce the putative intercellular communication through the analysis of ligand-receptor signaling pathways, we found that the overall number of upregulated and downregulated receptor-ligand pairs was similar in the high mTORC1 signaling group. However, it was observed that fibroblast-related receptor-ligand pairs were upregulated in this group (Fig. 6D). After analyzing the scRNA-seq data of seven bladder cancer patients, it was found that there is a significant change in receptor-ligand pairs in high mTORC1 signaling groups. Out of the 20 most significant changed receptor-ligand pairs, 14 pairs were related to fibroblasts and were upregulated (Fig. 6E). This indicated that both anti-tumour immune and immune escape pathways were activated and counteract each other in high mTORC1 signaling group patients. Furthermore, tumor-associated fibroblasts might play an important role in this process.

### Potential of mTORC1 signaling for predicting therapeutic opportunities

**Figure 7:**
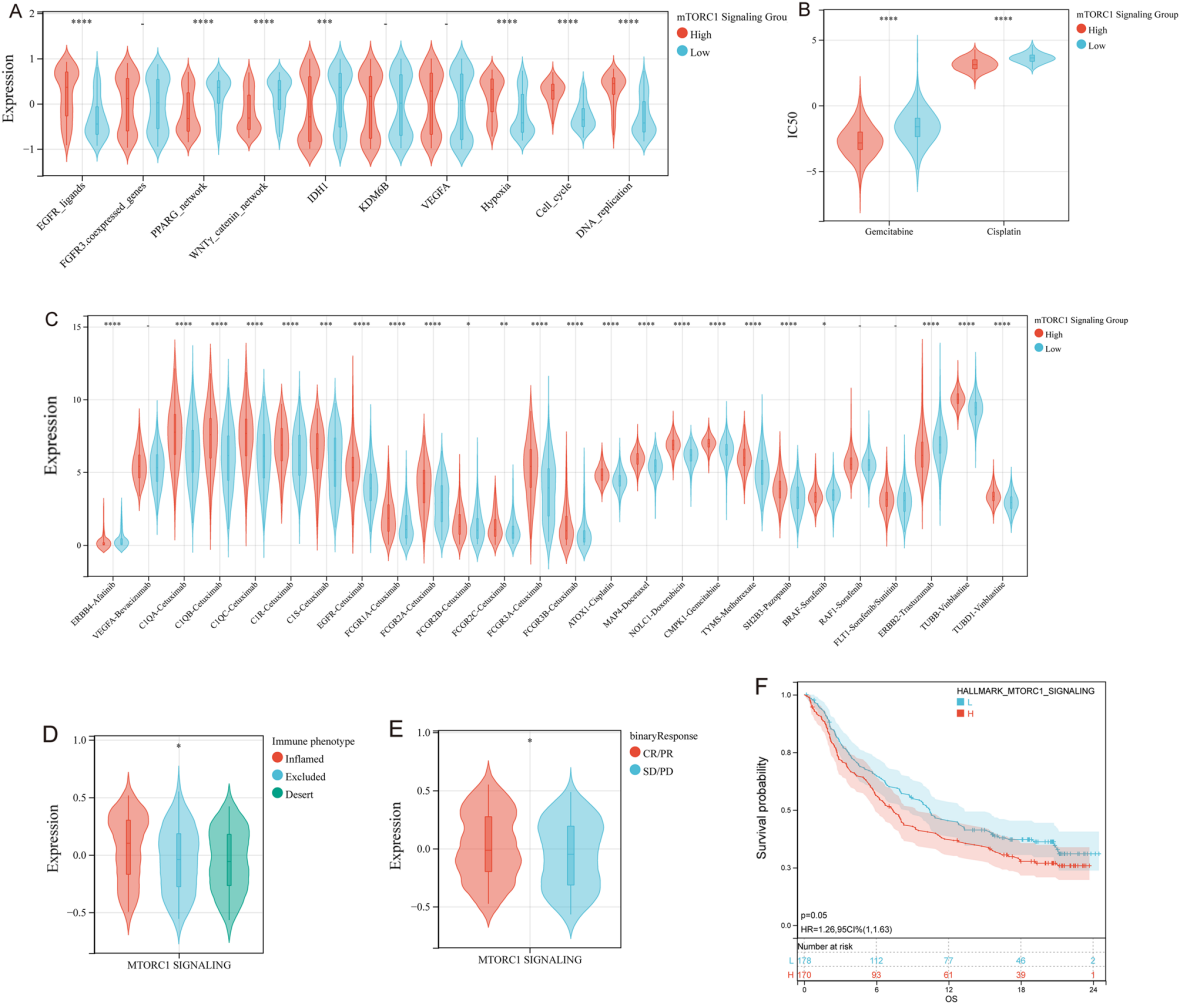
Correlation between mTORC1 and Therapeutic Response in Bladder Cancer. (**A**–**C**) Differential expression of (**A**) enrichment scores of therapeutic signatures such as targeted therapy and radiotherapy, (**B**) IC50 values of gemcitabine and cisplatin, and (**C**) drug-target genes in patients with high and low mTORC1 scores. (**D**, **E**) mTORC1 scores in patients with different (**D**) immune phenotypes and (**E**) clinical response to cancer immunotherapy in the IMvigor210 cohort. (**F**) Kaplan–Meier analysis of mTORC1 scores in the IMvigor210 cohort.

In this study, we further investigated the relationship between mTORC1 signaling and the response to chemotherapy, radiotherapy, and targeted therapies. Our analysis revealed that the high mTORC1 signaling group exhibited higher scores for the network associated with epidermal growth factor receptor (EGFR) and its ligands, and pathways associated with radiotherapy response (cell cycle, DNA replication, and hypoxia). Whereas, the low mTORC1 signaling group demonstrated higher scores for immune-suppressive oncogenic pathways (PPARG network, WNTγ catenin network, and IDH1) (Fig. 7A).

Gemcitabine and cisplatin are two of the most frequently utilized chemotherapy drugs in the treatment of bladder cancer. In this study, we calculated the IC_50_ values for these agents using pRRophetic, and found that the high mTORC1 signaling group demonstrated greater sensitivity to both drugs (Fig. 7B). We also analyzed the Drugbank database, which revealed that the high mTORC1 signaling group expressed higher EGFR inhibitors and targets related to chemotherapeutic agents. These findings suggest that the high mTORC1 signaling group may respond better to these chemotherapeutic agents and may benefit from treatment with EGFR inhibitors (Fig. 7C). Furthermore, the analysis of the IMvigor210 cohort data showed that the patients who had higher mTORC1 signaling scores had a greater number of immune cells infiltrating their tumors (Fig. 7D). Additionally, patients who exhibited a better response to immunotherapy had higher mTORC1 signaling scores (Fig. 7E). However, despite demonstrating better responses to immunotherapy, patients with higher mTORC1 signaling scores had worse prognoses (Fig. 7F). These findings suggest that the role of mTORC1 signaling in the tumor microenvironment is complex and may have different effects on the response to various therapies.

We conducted a mutation profile analysis of the TCGA-BLCA cohort and found that RYR3, FAT3, and VCAN were the three most significantly mutated genes (Supplementary Fig. S5). The mutation frequency of RYR3 was higher in the low mTORC1 signaling group, whereas FAT3 and VCAN had higher mutation frequencies in the high mTORC1 signaling group.

### mTORC1RS development and validation

**Figure 8:**
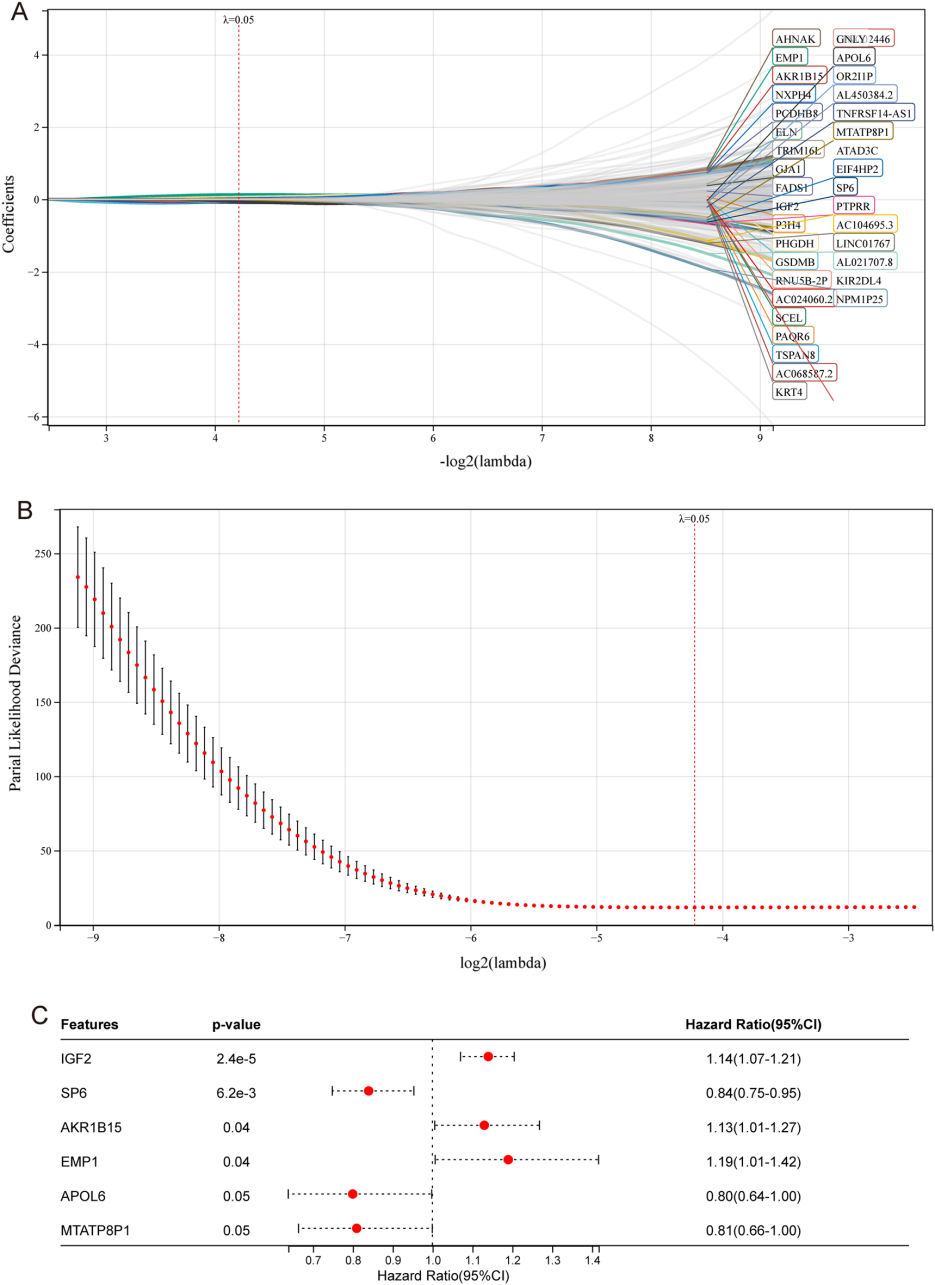
Construction of the mTORC1RS. (**A**, **B**) Candidate genes were screened using the LASSO regression method. (**C**) Multivariate Cox regression was performed to identify key genes.

**Figure 9:**
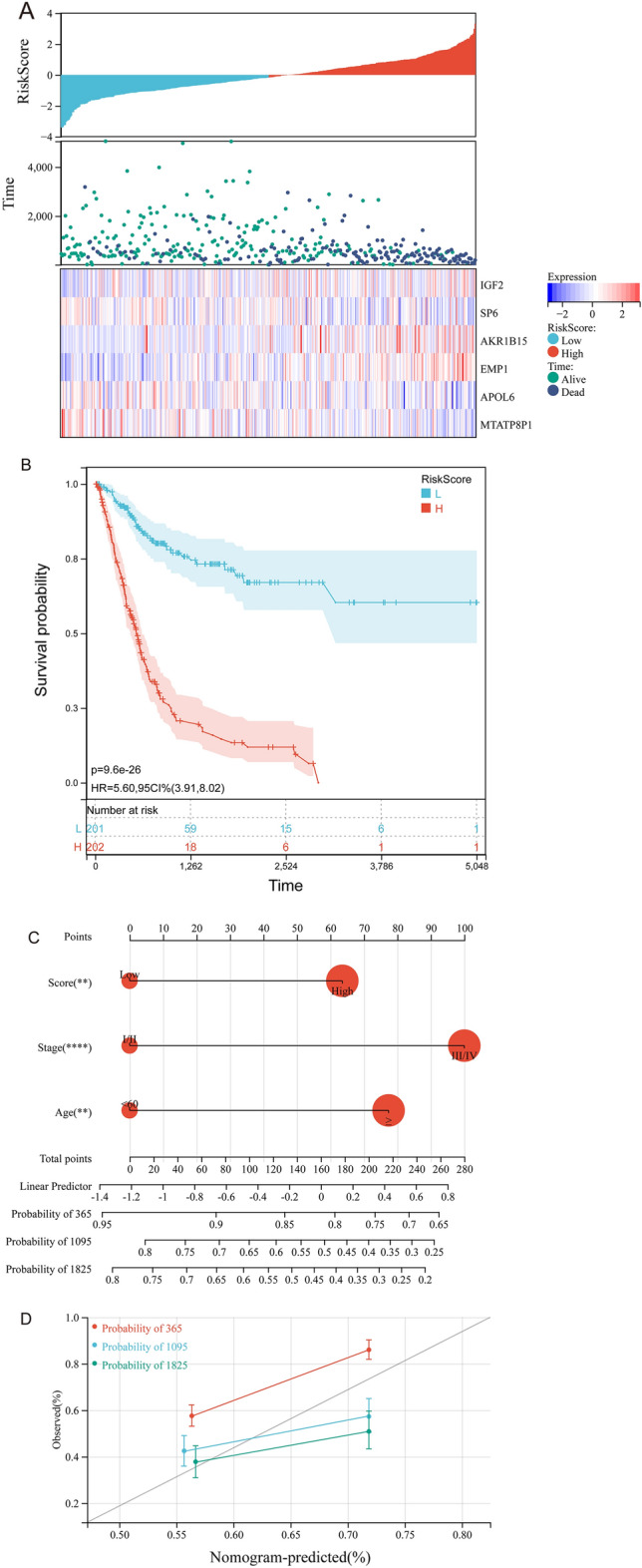
Validation of the mTORC1RS and establishment of the mTORC1RS related nomogram. (**A**, **B**) Analysis of risk scores and Kaplan–Meier analysis of mTORC1RS in the TCGA cohort. (**C**) Nomogram for predicting overall survival probability using age, mTORC1RS group, and tumor stage as parameters. (− *P *> 0.05, *P < 0.05, **P < 0.01, ***P < 0.001, and ****P < 0.0001 for multivariate regression of clinical factors and mTORC1RS). (**D**) Calibration curves to verify the accuracy of predictions, where red represents the 1-year prediction, blue represents the 3-year prediction, and green represents the 5-year prediction.

To establish the mTORC1RS, we first conducted univariate Cox regression on 3,001 genes associated with OS that were identified from DEGs between high and low mTORC1 signaling groups. To prevent overfitting, we applied LASSO regression analysis (Fig. 8A,B) and multivariate Cox regression (Fig. 8C) to further refine the model. Eventually, six genes were identified to construct the mTORC1RS: EMP1 (weight = 0.17755), AKR1B15 (weight = 0.12164), SP6 (weight =  − 0.1685), MTATP8P1 (weight =  − 0.2049), APOL6 (weight =  − 0.2254), and IGF2 (weight = 0.12738). Using the mTORC1RS, patients were categorized into subgroups of high- and low-risk based on the median value of the score (Fig. 9A), and the predictive performance of the mTORC1RS was validated using KM survival curves (Fig. 9B). Notably, patients in the high mTORC1RS risk group had a significantly lower survival probability in the TCGA-BLCA cohort.

To develop a clinically applicable method for predicting patient survival probability, we constructed a nomogram that considered clinicopathological covariates. Based on the results of the Cox analysis, a nomogram was established to predict OS rates (Fig. 9C). Furthermore, calibration curves demonstrated that the nomogram linked to mTORC1RS accurately predicted the survival probability (Fig. 9D).

## Discussion

Bladder cancer is a prevalent disease that leads to high rates of morbidity and mortality, with recurrence and metastasis posing significant treatment challenges^1,2^. One key aspect of successful treatment is the identification of outcome-associated biomarkers and response to therapy. We aimed to explore the molecular landscape and phenotypic characteristics of bladder cancer with abnormal mTORC1 signaling and determine its potential influence on clinical outcomes and the immune microenvironment. The results of this investigation could aid in the development of individualized therapeutic approaches for bladder cancer patients.

New technologies such as transcriptome, genome, and bioinformatics have significantly contributed to the discovery of biomarkers that can inform personalized therapeutic interventions for cancer patients^25–28^. Our study revealed that mTORC1 signaling was overactivated in bladder cancer and correlated with poor prognosis. Our findings suggested that mTORC1 signaling could be used as an independent predictor of clinical outcomes for bladder cancer patients. Moreover, the study also revealed that mTORC1 dysregulation is associated simultaneously with antitumor immune activation and the activation of immune evasion mechanisms, highlighting the potential of mTORC1 in regulating the immune response in bladder cancer. Prior research has indicated that mTORC1 inhibitors like rapamycin can improve the immune response of T cells, which includes increased interferon-γ and cytokine production, T cell differentiation, and activation^29,30^. Therefore, targeting mTORC1 signaling could have therapeutic benefits for bladder cancer. This is because it could directly impede the growth of the tumor and also change the immune microenvironment by modulating the immune-related gene expression.

Besides, the findings of this study reveal a positive correlation between elevated mTORC1 signaling pathway scores and heightened mutation frequencies in FAT3 and VCAN. Concurrently, a negative association is demonstrated with the mutation frequency of RYR3. FAT3 and VCAN genes are intricately involved in processes such as cell adhesion, migration, and tumor invasion, all of which constitute pivotal aspects of cancer progression and metastasis^31,32^. These revelations suggest that patients with heightened mTORC1 activation might be more susceptible to developing a subtype of bladder cancer characterized by increased invasiveness and malignancy. The RYR3 gene encodes a calcium ion channel protein, and mutations in this gene may be linked to the dysregulation of calcium in tumor cells. Previous studie has reported that the inactivation of RYR3 results in constrained growth of breast cancer cells.^33^. Therefore, these results imply that the lower mutation frequency of the RYR3 gene in patients with high mTORC1 activation could signify a potential anti-cancer mechanism.

We identified six mTORC1-related genes and incorporated them into the mTORC1RS. Among these genes, EMP1, AKR1B15, and IGF2 were associated with high risk, while SP6, MTATP8P1, and APOL6 were associated with low risk. EMP1^34^ and IGF2^35^ have been found to promote tumor growth and metastasis, while APOL6 has been found to inhibit the migration and invasion of cancer cells^36^. However, there is currently no research on the role of AKR1B15, MTATP8P1, and SP6 in cancer. AKR1B15 is a type of aldo–keto reductase that is similar to AKR1B10 in terms of amino acid sequence, with a 92% identity^37^. AKR1B10 is an extensively studied enzyme with high retinaldehyde reductase activity linked to the development of various cancers. On the other hand, SP6 is a member of the transcription factor, characterized by three zinc finger domains for DNA binding containing Cys2His2 motif tetrahedrally coordinated with zinc atoms. These transcription factors attach to GC-rich motifs and associated GT and CACCC elements, and SP6 is no exception to this^38^. Additionally, MTATP8P1 is a pseudogene. Pseudogenes were initially thought to be non-functional genomic relics resulting from mutations in genes during evolution. However, subsequent research has shown that they play diverse roles at multiple levels (DNA, RNA, and/or protein) in various physiological and pathological processes, particularly in cancer, both dependent and independent of parent genes^39^. Given this evidence, further research is needed to investigate the role of AKR1B15, MTATP8P1, and SP6 in cancer.

Our study also has some limitations that need to be addressed. First, we only focused on the dysregulation of mTORC1 signaling in bladder cancer and did not investigate other pathways that may contribute to bladder cancer development and progression. Future studies should explore the interplay between mTORC1 signaling and other cellular pathways to obtain a more comprehensive understanding of bladder cancer pathogenesis. Second, our study only utilized publicly available gene expression data, and additional validation studies are needed to confirm our findings. Future studies should examine the correlation between mTORC1 signaling and immune cell infiltration to obtain a more comprehensive understanding of the immune microenvironment in bladder cancer.

In conclusion, our study provides new insights into the role of mTORC1 signaling in bladder cancer pathogenesis. Our identification of mTORC1-related genes associated with mTORC1 signaling dysregulation in bladder cancer may provide new opportunities for the development of personalized therapies targeting the mTORC1 signaling pathway involved in bladder cancer. However, further studies are needed to validate our findings and to explore the interplay between mTORC1 signaling and other cellular pathways involved in bladder cancer development and progression.

### Supplementary Information

Supplementary Figures.

Supplementary Tables.

## Data Availability

The raw data of our study were downloaded from TCGA dataset (http://cancergenome.nih.gov/), GEO dataset (GSE135337, https://www.ncbi.nlm.nih.gov/geo/query/acc.cgi?acc=GSE135337) and IMvigor210CoreBiologies dataset (http://research-pub.gene.com/IMvigor210CoreBiologies/).

## Abbreviations

mTORC1: Mammalian target of rapamycin complex 1
Raptor: Regulatory-associated protein of mTOR
mLST8: Mammalian lethal with Sec13 protein 8
PRAS40: Proline-rich Akt substrate of 40 kDa
DEPTOR: DEP-domain-containing mTOR-interacting protein
S6K1: S6 kinase 1
4EBP1: 4E-binding protein 1
RNA-seq: RNA sequencing
FPKM: Fragments per kilobase of exon per million mapped fragments
TCGA: The Cancer Genome Atlas
TPM: Transcripts per million
MAF: Mutation annotation format
scRNA-seq: Single-cell RNA-seq
GSVA: Gene set variation analysis
MsigDB: Molecular signatures database
DEGs: Differentially expressed genes
FC: Fold change
GO: Gene ontology
KEGG: Kyoto Encyclopedia of Genes and Genomes
GSEA: Gene set enrichment analysis
IC50: Half-maximal inhibitory concentration
mTORC1RS: MTORC1-related signature
OS: Overall survival
KM: Kaplan–Meier
EMT: Epithelial-mesenchymal transition
TIME: Tumor immune microenvironment
EGFR: Epidermal growth factor receptor
