# Supplementary material for: Investigating the prognostic value of mTORC1 signaling in bladder cancer via bioinformatics evaluation
Source: Sci Rep. 2023 Dec 12;13:22066. doi: 10.1038/s41598-023-49366-w (PMC10716140; doi:10.1038/s41598-023-49366-w)

Supplementary Fig. S1.Heatmap of mTORC1 gene expression in normal and bladder cancer tissues.

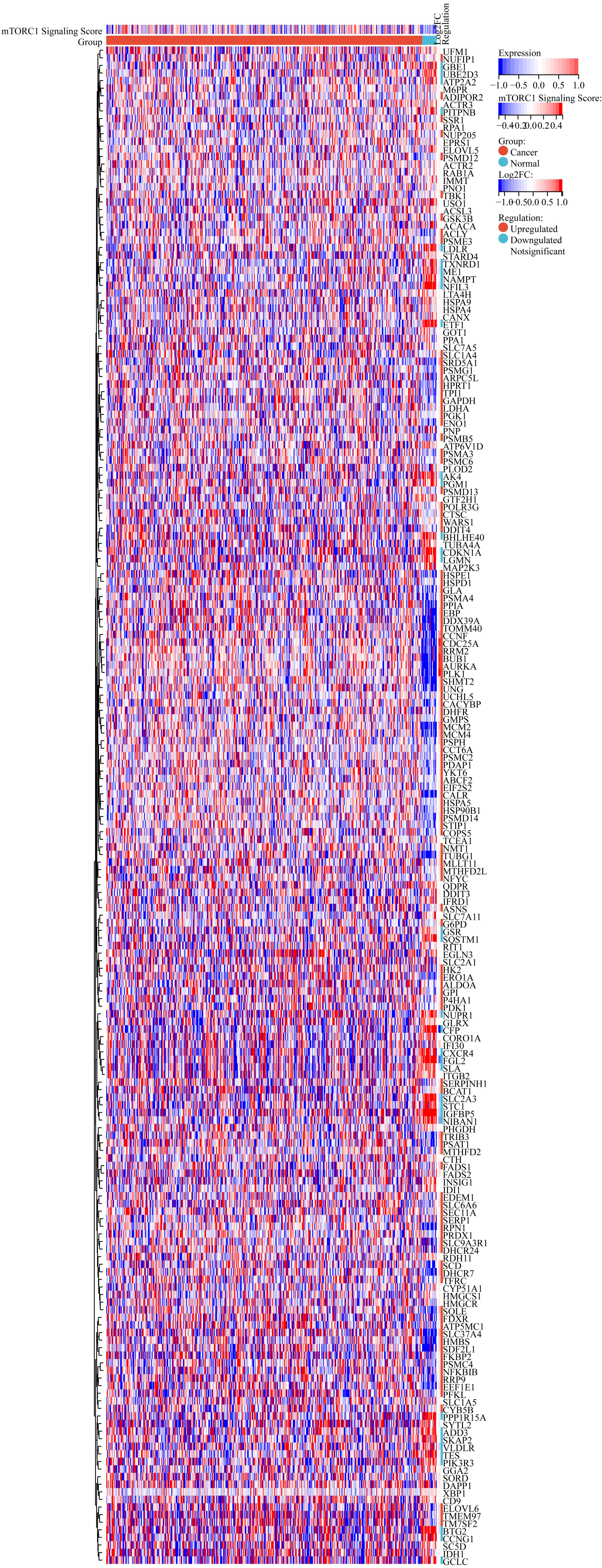

Supplementary Fig. S2.Risk score analysis for mTORC1 score in OS of bladder cancer.

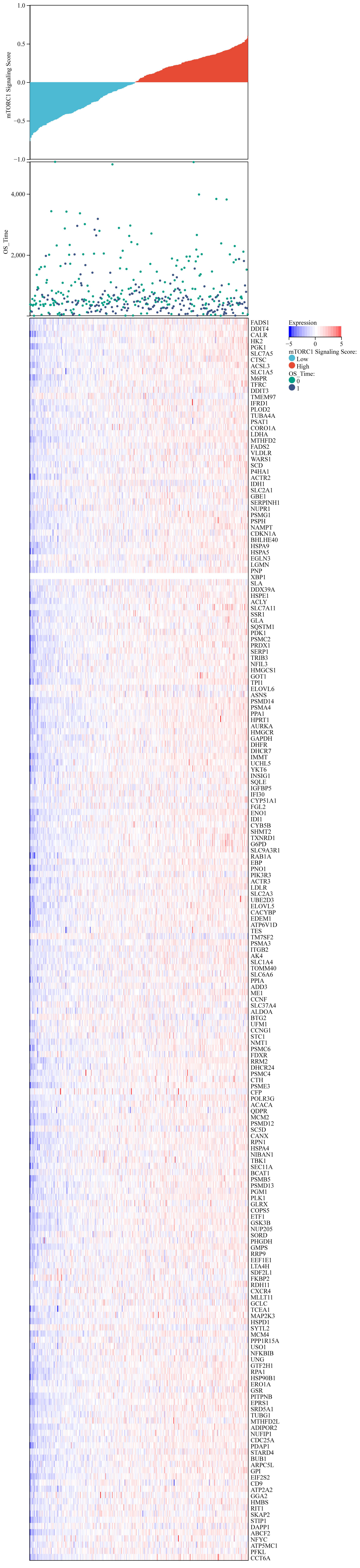

Supplementary Fig. S3. Identification of DEGs between the mTORC1 groups.
(A) Volcano plot of DEGs. (B) Heatmap of DEGs; the change from red to blue represents a gradual decrease in relative gene expression.

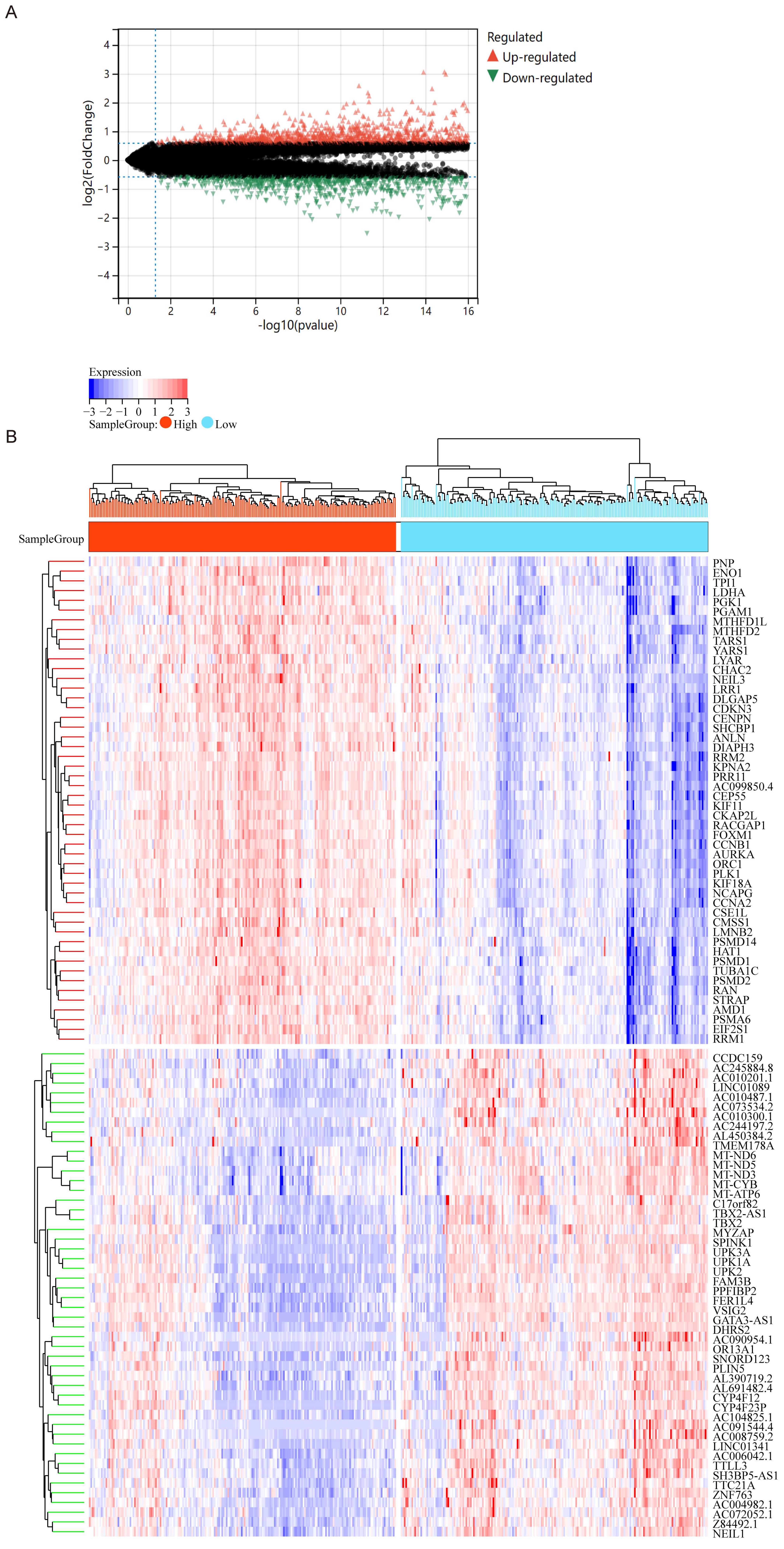

Supplementary Fig. S4. Correlation analysis between mTORC1 and tumor immune microenvironment (TIME). (A-C) Correlation heatmap of (A) immune cells, (B) immune checkpoints, and (C) immune cycle score with mTORC1 score in bladder cacner.(D) t-SNE plot showing the composition of 6 main cell types. (E) (C) Distribution in the mTORC1 of seven bladder cancer samples (divided into 2 patterns).

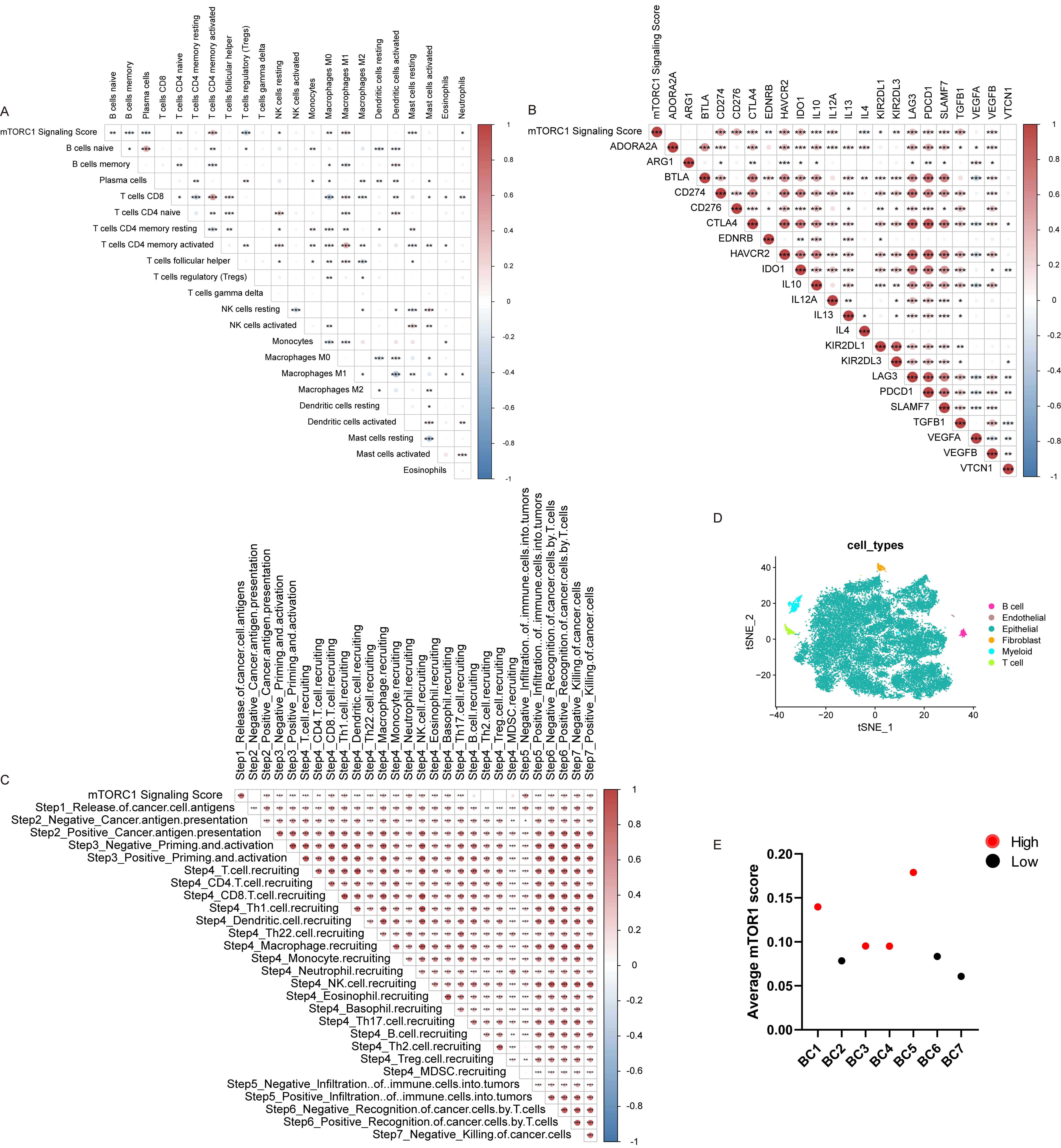

Supplementary Fig. S5. Comparison of the mutation landscape between groups with high and mTORC1 score.

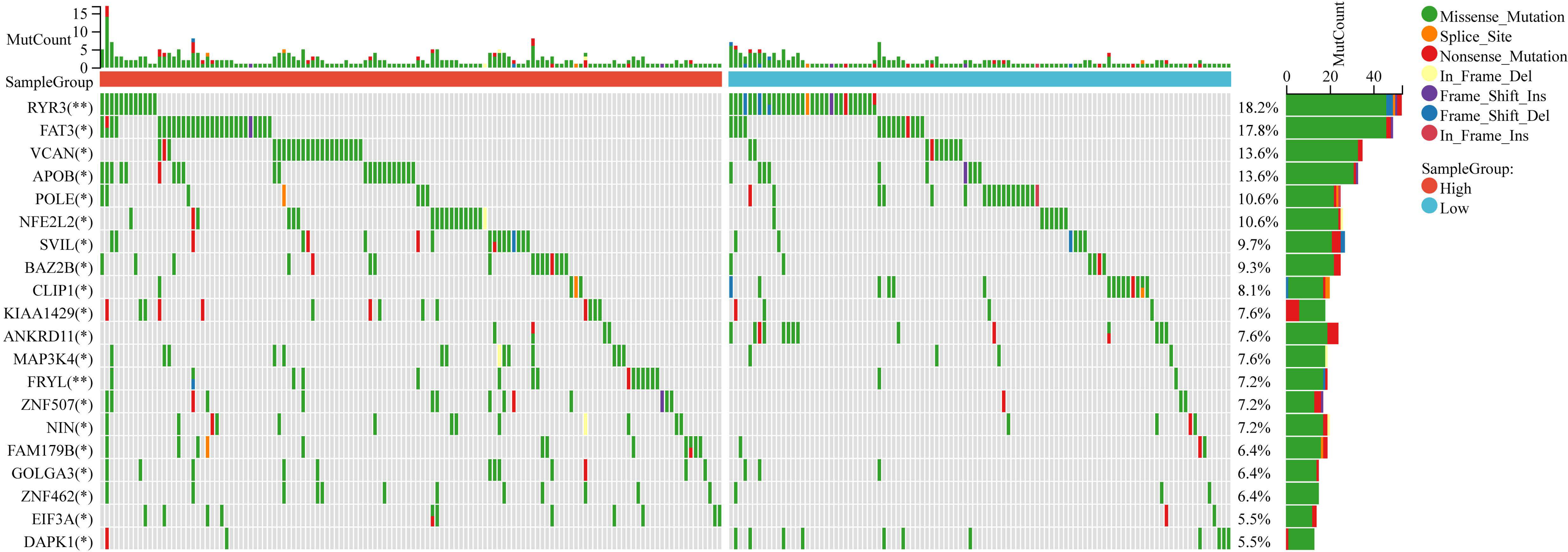

Supplement: Supplementary file 1 — Supplementary Figures. [file 41598_2023_49366_MOESM1_ESM.pdf]
